# Origin and Expansion of the Yunnan Shoot Borer, *Tomicus yunnanensis* (Coleoptera: Scolytinae): A Mixture of Historical Natural Expansion and Contemporary Human-Mediated Relocation

**DOI:** 10.1371/journal.pone.0111940

**Published:** 2014-11-05

**Authors:** Jun Lü, Shao-ji Hu, Xue-yu Ma, Jin-min Chen, Qing-qing Li, Hui Ye

**Affiliations:** 1 Laboratory of Biological Invasion and Ecosecurity, Yunnan University, Kunming, 650091, China; 2 Yunnan Key Laboratory of International Rivers and Transboundary Eco-security, Yunnan University, Kunming, 650091, China; 3 School of Mathematics and Computer Science, Yunnan University of Nationalities, Kunming, 650031, China; 4 Laboratory for Conservation and Utilization of Bio-resources, Yunnan University, Kunming, 650091, China; 5 Life Science College, Yunnan Normal University, Kunming, 650092, China; University of Innsbruck, Austria

## Abstract

The Yunnan shoot borer, *Tomicus yunnanensis*, is a recently-discovered, aggressive pest of the Yunnan pine stands in southwestern China. Despite many bionomics studies and massive controlling efforts, research on its population genetics is extremely limited. The present study, aimed at investigating the origin and dispersal of this important forestry pest, analyzed the population genetic structure and demographic history using a mitochondrial *cox1* gene fragment. Our results showed that *T. yunnanensis* most likely originated from the Central-Yunnan Altiplano, and the divergence time analysis placed the origin approximately 0.72 million-years ago. Host separation and specialization might have caused the speciation of *T. yunnanensis*. Genetic structure analyses identified two population groups, with six populations near the origin area forming one group and the remaining six populations from western and eastern Yunnan and southwestern Sichuan comprising the other. Divergence time analysis placed the split of the two groups at approximately 0.60 million-years ago, and haplotype phylogenetic tree, network, as well as migration rate suggested that populations of the latter group were established via a small number of individuals from the former one. Migration analysis also showed a certain degree of recent expansion from southwestern Sichuan to eastern Yunnan. Our findings implied that *T. yunnanensis* underwent both historical expansion and recent dispersal. The historical expansion may relate to the oscillation of regional climate due to glacial and interglacial periods in the Pleistocene, while human-mediated transportation of pine-wood material might have assisted the relocation and establishment of this pest in novel habitats.

## Introduction

The Yunnan shoot borer, *Tomicus yunnanensis* Kirkendall & Faccoli (Coleoptera: Curculionidae: Scolytinae), is one of the most aggressive pest species in genus *Tomicus*, which has caused serious annual damage in up to 20,000 ha of the Yunnan pine, *Pinus yunnanensis* Franchet, since the major outbreak in southwestern China in the 1980s [1]–[3].

For the past two decades, *T. yunnanensis* has been confused with *T. piniperda* Linnaeus due to morphological resemblance, but genetic comparison between the southwestern Chinese population and the Eurasian *T. piniperda* suggested that the former should be treated as a previously undescribed *Tomicus* species [4]. Based on these findings, Kirkendall *et al*. (2008) analyzed the morphological characters of all known *Tomicus* species and described this beetle for the first time [5].

The Yunnan pine, *P. yunnanensis*, is the only known host of *T. yunnanensis* to date [3]. This pine is the most important silvicultural tree in southwestern China due to its high tolerance to drought [6]. Therefore, the continuous infestation and constant outbreak of *T. yunnanensis* has brought severe damage to the environment in this region [7]. *T. yunnanensis* is endemic to southwestern China, and its distribution range is highly overlapped with the Yunnan pine [3]. The first population outbreaks of this beetle were recorded in the Central-Yunnan altiplano, and then in northern, western, and southern Yunnan in the following years [8]–[10]. Since 2000, the beetle has been reported in the Yunnan pine stands in southwestern Sichuan (i.e., Liangshan Prefecture) and western Guizhou (i.e., Panxian County) [11], [12].

The origin of *T. yunnanensis* and the formation of its current distribution pattern are two interesting questions underpinning the infestation process of this pest. In an attempt to answer these questions, the present study used mitochondrial DNA data to analyze the genetic structure, population relationships, and demographic history of *T. yunnanensis*. The present study will elucidate the origin of the species, and understand its historical expansion routes. Meanwhile, the findings may also benefit the quarantine procedures to prevent further expansion of this pest.

## Materials and Methods

### Sample Collection

**Figure 1:**
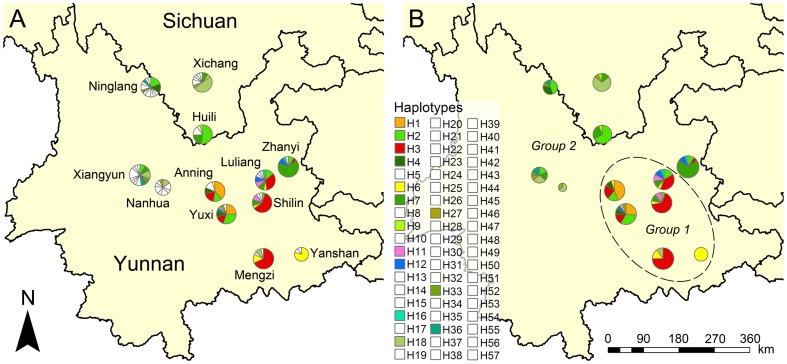
Haplotype distribution of the 12 populations of *T. yunnanensis*, (A) distribution of all haplotypes with private haplotypes marked in white and (B) distribution of only shared haplotypes and the tentative boundary (dash line) between the two population groups defined by SAMOVA, the circle sizes corresponding to the sample size of each population.

In total, 231 individuals were collected representing 12 populations of *Tomicus yunnanensis* (10 populations from Yunnan and two from Sichuan), with the sample size of the population from Yanshan being the lowest (10 individuals) and that of the population from Xiangyun being the greatest (23 individuals). The geographical coordinates and altitudes of sampling sites were recorded with a Garmin eTrex Vista GPS handset (Version 3.2; Garmin Ltd., Taiwan) (Table 1; Fig. 1).

**Table 1:** Summary information of 12 sampling sites, arranged by ascending order of locality code.

Code	Locality	Coordinate	Alt./m	Time	Size
AN	Yunnan: Anning	24.97, 102.33	1,880	Jan. 2011	21
HL	Sichuan: Huili	26.30, 102.05	2,164	Aug. 2011	19
LL	Yunnan: Luliang	25.15, 103.66	1,883	Jan. 2011	21
MZ	Yunnan: Mengzi	23.46, 103.44	1,891	Jul. 2011	22
NH	Yunnan: Nanhua	25.09, 101.14	1,899	Mar. 2011	13
NL	Yunnan: Ninglang	27.38, 100.86	2,490	Aug. 2007	20
SL	Yunnan: Shilin	24.74, 103.41	1,903	Jan. 2011	20
XC	Sichuan: Xichang	27.49, 102.04	1,700	Aug. 2011	20
XY	Yunnan: Xiangyun	25.37, 100.61	2,257	Mar. 2011	23
YS	Yunnan: Yanshan	23.56, 104.31	1,566	Jul. 2011	10
YX	Yunnan: Yuxi	24.48, 102.59	1,703	Mar. 2011	20
ZY	Yunnan: Zhanyi	25.56, 104.01	2,132	Mar. 2011	22

Adult beetles were captured from egg galleries on trunks of *Pinus yunnanensis* in 2007 to 2012 and instantly preserved in 95% ethanol. In order to avoid sampling closely related individuals, for instance individuals sharing the same parents, at least five pine trees 100 m apart were chosen to collect the beetles, and the samples used in the present study were randomly taken from the mixed sample pool of each population.

An individual of *T. armandii* Li & Zhang and an individual of *T. piniperda* Linnaeus were chosen as outgroup due to their close phylogenetic lineage to *T. yunnanensis* (Ma *et al*. unpublished data). All samples were carefully identified under a Nikon SMZ1500 stereoscope (Nikon, Japan) following Kirkendall *et al*. (2008) [5] and Li *et al*. (2010) [13]. Selected samples were stored at −40°C in the Laboratory of Biological Invasion and Ecosecurity, Yunnan University until DNA extraction.

### Ethics Statement

No specific permits were required in the present study for collection of this native forest insect. The authors confirm that the sampling sites were not privately owned or protected. This study did not involve any endangered or protected species.

### Genomic DNA Extraction

In order to prevent contamination by parasitoids and fungi, each sample was washed twice with double distilled water, and the antennae, elytra, and abdomen were removed [4]. Samples were individually treated with 1 mL STE solution at room temperature for 24 h prior to DNA extraction to eliminate the residual ethanol and to rehydrate the tissue.

Endosymbiotons such as *Wolbachia* and the nuclear copies of mtDNA fragments (*Numts*) are the most common problems in phylogenetic research using mtDNA markers [14]–[17]. To minimize the possibility of such pitfall, the mesothoracic muscles containing high density of mitochondria were extracted for DNA extraction to reduce the possibility of obtaining *Numts* in PCR, and a *Wolbachia* search was also carried out to detect possible contamination (see below). Extraction protocol for genomic DNA followed the phenol-chloroform method described by Hu *et al*. (2013) [18]. Product DNA were quantified on an Eppendorf Biophotometer (Eppendorf AG, Hamburg, Germany), and preserved at −40°C in the same laboratory. The ≈100 ng/*µ*L dilutions were used as templates in polymerase chain reactions (PCR).

### PCR Amplification and Sequencing

For all samples, a ≈800 bp fragment of the *cox1* gene was amplified by PCR on a Biometra T-Professional Standard thermocycler (Biometra GmbH, Göttingen, Germany). The thermal profile consisted of an initial denaturation at 94°C for 3 min; followed by 35 cycles of denaturation at 94°C for 30 sec, annealing at 47°C for 1 min, and elongation at 72°C for 2 min; then a final elongation at 72°C for 10 min.

The PCR reaction was applied in a 25* µ*L volume system using the TaKaRa Ex *Taq* kit (TaKaRa Biotechnology Co., Ltd., Dalian, China), which contained 2.5* µ*L of 10× PCR buffer, 2.5* µ*L of MgCl_2_ (25 mmol/L), 4.0* µ*L of dNTP mixture (2.5 mmol/L each), 0.5* µ*L of both the forward and reverse primers (20* µ*mol/L; Shanghai Sangon Biological Engineering Technology & Services Co., Ltd., Shanghai, China), 0.25* µ*L of Ex *Taq* polymerase (5 U/*µ*L), and 1.0* µ*L of DNA template. The primers used to amplify the fragment were Cl-J-2183 (5′-CAA CAT TTA TTT TGA TTT TTT GG-3′) and T2-N-3014 (5′-TCC AAT GCA CTA ATC TGC CAT ATT A-3′) [19].

In order to finally exclude the possibility of obtaining *Wolbachia* genes in the PCR products, a strict *Wolbachia* search was also carried out. Another set of PCR was applied to all samples for the *Wolbachia* surface protein (*wsp*) gene using primers *wsp* 81F (5′-TGG TCC AAT AAG TGA TGA AGA AAC-3′) and *wsp* 691R (5′-AAA AAT TAA ACG CTA CTC CA-3′) [20], and the PCR thermal profile and reaction system followed Braig et al. (1998) [20]. The 1% agarose gel electrophoresis was used to determine the presence and size of the *wsp* gene. The genomic DNA extracted from the abdomen of a *Wolbachia* infected female *Sogatella furcifera* (Horváth) (Hemiptera: Delphacidae) and a tetracycline-treated female *S. furcifera* were used as positive and negative controls respectively.

The PCR products of *cox1* gene were purified using TaKaRa Agarose Gel Purification Kit (version 2.0) (TaKaRa Biotechnology Co., Ltd.) and sequenced by Sangon Biological Engineering Technology & Services Co., Ltd. (Shanghai, China). Sequencing reactions were carried out in both forward and reverse directions on an ABI Prism 3730xl automatic sequencer (Applied Biosystems, Foster City, CA, USA).

### Data Analyses

#### Sequence alignment

Raw sequences were proofread and aligned using Clustal W [21] in BioEdit 7.0.9 [22], and any sequence containing double peaks in the chromatograms was strictly excluded. The product sequences were checked by MEGABLAST against the genomic references (refseq_genomic) and nucleotide collection (nr/nt) in NCBI, as well as conceptual translation using the invertebrate mitochondrial criterion in MEGA 5.1 [23] to detect possible *Numts* (nuclear copies of mtDNA fragments). Also, a search for nonsynonymous mutations, in-frame stop codons, and indels was carried out to further minimize the existence of cryptic *Numts* [15], [17]. Number of polymorphic sites and nucleotide composition were analyzed in MEGA 5.1. Haplotypes were defined by DnaSP 5.0 [24], and the ratios of shared and private haplotypes were calculated respectively.

#### Genetic distance and diversity indices

Genetic distances and standard errors between populations were calculated with Kimura's two-parameter (K2P) model [25] in MEGA 5.1 with 1,000 iterations. Nei's average number of pairwise differences between populations [26], Nei's average number of pairwise differences within populations, pairwise *F*st values, haplotype diversity (*H*) and nucleotide diversity (*π*) of each population, the degree of gene flow among populations (*N*_m_) were calculated in Arlequin 3.11 [27]. The significance of Nei's average number of pairwise differences between populations and the pairwise *F*st values were tested by 1,000 iterations for statistical significance, and the optimal gamma shape used in Arlequin 3.11 was estimated by jModelTest 0.1 [28], [29].

#### Population genetic structure and phylogeny

A multidimensional scaling (MDS) plot [30] was drawn from the K2P distance in SPSS 17.0 (SPSS Inc., Chicago, IL, USA) to analyze the genetic relationship between the 12 populations of *T. yunnanensis*. To analyze the genetic structure of the 12 populations of *T. yunnanensis*, the spatial analysis of molecular variance (SAMOVA) was performed in SAMOVA 1.0 [31] by setting different numbers of groups (*K*) to the dataset. The optimal *K* value was selected when the *F*_CT_ value in the training results reached the plateau and no single population was assigned to any group. In the present study, the range of *K* was 2–6 in the training. Once the optimal *K* value was obtained, the result of SAMOVA analysis was tested by the analysis of molecular variance (AMOVA) [32] with 1,000 iterations in Arlequin 3.11. The above-mentioned genetic distances and the *N*_m_ value between the population groups were calculated using MEGA 5.1 and Arlequin 3.11. A median-joining haplotype network was constructed using Network 4.6 (Fluxus Engineering Ltd.) and categorized by population groups determined by the SAMOVA analysis.

A phylogenetic tree of haplotypes was reconstructed using the Bayesian Inference (BI) method using MrBayes 3.2.2 [33], with the most appropriate nucleotide substitution model estimated by jModelTest 0.1 [28], [29]. A 1,500,000 step of MCMC (Markov chain Monte Carlo) replications was applied for the Bayesian Inference in an attempt to obtain an average standard deviation of split frequency below 0.01, and a 25% burn-in criterion was used to summarize the tree [33]. The resultant tree was edited and annotated in FigTree 1.4 [34]. Molecular clock is an effective tool for inferring divergence time when fossil evidence is unavailable [35]. The divergence time was estimated using *t*_MRCA_ (time to most recent common ancestor) in BEAST 1.7.5 [36] with 10,000,000 steps of MCMC. The substitution rate for the *t*_MRCA_ inference was calibrated at 1.03% per million years for Polyphaga [37].

#### Demographic history and isolation-by-distance (IBD)

In an attempt to analyze the demographic history of *T. yunnanensis*, the mismatch distributions [38] of each population as well as population groups were calculated in Arlequin 3.11 to detect past population expansion, as long-time stable populations show a multimodal curve while the expanded ones usually generate unimodal curves [39]. Significant sum squared deviations (SSD) and raggedness index were used to reject the rapid expansion model as expanded populations are expected to exhibit smaller values of SSD and raggedness index. Neutrality test of Tajima's *D* [40] and Fu's *F_s_* [41] were also examined in Arlequin 3.11 with 1,000 iterations to test statistical significance, as the significant negative values indicate genetic hitchhiking, which might be caused by some events in demographic history (i.e., recent expansion, background selection, or genetic sweeping).

The software Migrate 3.5.1 was used to assess the degree of genetic exchange between populations by estimating the migration rates from one population to the another using both of the Bayesian inference and the maximum likelihood methods [42]. For Bayesian inference, three long chains were applied with 100,000,000 steps of MCMC each and 10,000 steps of burn-in; for maximum likelihood, 10 short chains and three long chains were applied with 1,000,000 steps and 100,000,000 steps, respectively. The combination of its analytic result and the previously obtained *N*_m_ values can be finally used to determine the extent of genetic exchange between populations.

The isolation-by-distance (IBD) is an effective tool for detecting the correlation between genetic and geographic distance. A null hypothesis was tested using the pairwise genetic differentiation *F*st against the pairwise geographic distances (as well as the logarithm transformation) between populations. The approximate spherical distances between populations were measured between coordinates of each sampling locality in Google Earth. The test of IBD was performed using IBDWS 3.23 [43] with 10,000 iterations for Mantel test to determine the correlation between the genetic and geographic distances. The extent of this correlation was then determined by the regression of *F*st against the geographic distance and its logarithm transformation.

## Results

### Sequence Variability and Haplotype Distribution

The proofread of all chromatograms did not find any double peaks. The *Wolbachia* search detected only eight infected samples scattered in five populations, and no sign of correlation between the infection rate and the molecular diversity indices was found (Fig. S1; Table 2). The alignment yielded a 720 bp *cox1* fragment, which correspond to the 2,247–2,966 bp portion in the mitogenome of *Drosophila melanogaster* Meigen. The sequence contained 41 polymorphic sites (5.7%), 28 informative parsimony sites (3.9%), and 13 singleton variation sites (1.8%). No indel and in-frame stop codon were detected. Based on current available analytical tools, no sign of *Numts* was detected. The nucleotide composition showed a high A-T bias of nucleotide usage comprising 69.3% of the total average nucleotide composition.

**Table 2:** Haplotype distribution and the number of *Wolbachia* infected samples (*N_Wol_*) in the 12 populations of *T. yunnanensis*, the private haplotypes were underlined.

Population	Haplotypes	*N_Wol_*
Anning	H1, H2, H3, H4, H5, H6	3
Huili	H2, H7, H8, H9, H10	0
Luliang	H2, H3, H6, H7, H11, H12, H13, H14, H15, H16	1
Mengzi	H3, H6, H17, H18, H19	2
Nanhua	H18, H20, H21, H22, H23, H24, H25, H26, H27	0
Ninglang	H2, H4, H28, H29, H30, H31, H32, H33, H34, H35, H36, H37, H38	0
Shilin	H2, H3, H6, H7, H11, H39, H40	1
Xichang	H7, H9, H18, H27, H41, H42, H43, H44, H45	0
Xiangyun	H2, H7, H18, H27, H33, H36, H46, H47, H48, H49, H50, H51, H52, H53	1
Yanshan	H6, H54, H55	0
Yuxi	H1, H2, H3, H4, H12, H18, H27, H56	0
Zhanyi	H2, H3, H7, H12, H16, H57	0

Fifty-seven haplotypes of the *cox1* sequence were defined based on polymorphic sites and designated in numeral order, among which 14 haplotypes (24.6%) were shared by at least two populations and the remaining 43 haplotypes (75.4%) were locally private. All haplotypes and outgroup were deposited in GenBank under the accession numbers JX448430–JX448477 (H1–H47 and *T. armandii*) and KC986943–KC986953 (H48–H57 and *T. piniperda*).

For each of the 12 populations of *T. yunnanensis*, the number of haplotypes varied greatly, with three haplotypes being the least in the population from Yanshan and 14 being the most abundant in the population from Xiangyun (Table 2; Table 3; Fig. 1). The haplotype distribution showed that 67.4% of the total number of private haplotypes was found in populations from Nanhua, Ningliang, Xichang, and Xiangyun. Meanwhile, five (62.5%) of the shared haplotypes in populations from Huili, Nanhua, Ninglang, Xichang, Xiangyun, and Zhanyi were also found in populations from Anning, Luliang, Mengzi, Shilin, and Yuxi.

**Table 3:** The haplotype diversity (*H*), nucleotide diversity (*π*), number of haplotypes (*h*), ratio of shared haplotypes (*R*_shr_), and ratio of private haplotypes (*R*_prv_) of the 12 populations of *T. yunnanensis* and the two population groups defined by SAMOVA and the global dataset.

Population	*H*	*π*	*h*	*R*_shr_	*R*_prv_
Anning	0.805±0.059	0.00585±0.00338	6	0.83	0.17
Huili	0.684±0.092	0.00454±0.00273	5	0.60	0.40
Luliang	0.871±0.057	0.00704±0.00397	10	0.70	0.30
Mengzi	0.528±0.118	0.00334±0.00210	5	0.60	0.40
Nanhua	0.949±0.042	0.00578±0.00345	9	0.22	0.78
Ninglang	0.942±0.034	0.00397±0.00244	13	0.31	0.69
Shilin	0.642±0.118	0.00506±0.00299	7	0.71	0.29
Xichang	0.753±0.094	0.00443±0.00267	9	0.44	0.56
Xiangyun	0.949±0.026	0.00497±0.00292	14	0.43	0.57
Yanshan	0.378±0.181	0.00139±0.00115	3	0.33	0.67
Yuxi	0.847±0.051	0.00605±0.00349	8	0.88	0.13
Zhanyi	0.537±0.123	0.00485±0.00287	6	0.83	0.17
Group 1	0.830±0.027	0.00586±0.00325	22	0.41	0.59
Group 2	0.921±0.015	0.00596±0.00329	43	0.16	0.84
Global	0.922±0.009	0.00726±0.00390	57	0.25	0.75

### Diversity Indices and Genetic Distances

The haplotype diversity (*H*) of the 12 populations of *T. yunnanensis* was 0.378–0.949, with the diversity index of the Yanshan population being the lowest (the sample size was also smaller) and that of the populations from Nanhua, Ninglang, and Xiangyun all exceeded 0.94 (Table 3). The nucleotide diversity (*π*) of the 12 populations varied from 0.00139 to 0.00704, with the index of the Yanshan population being the lowest and that of the Luliang population being the greatest (Table 3). The ratio of shared haplotypes (*R*_shr_) also varied among the 12 populations, with that of the population from Nanhua being the lowest and that of the population from Yanshan being the highest (Table 3). The sum of squared deviations (SSD) were not significant in 11 populations but was flagged with 0.01-level significance for the population from Mengzi; however, no significant value of raggedness index was detected among all 12 populations (Table 3).

The Kimura two-parameter (K2P) distances, *N*_m_ values, pairwise *F*st values, and Nei's average number of differences between populations were shown in Table S1 to S3.

### Population Genetic Structure and Phylogeny

**Figure 2:**
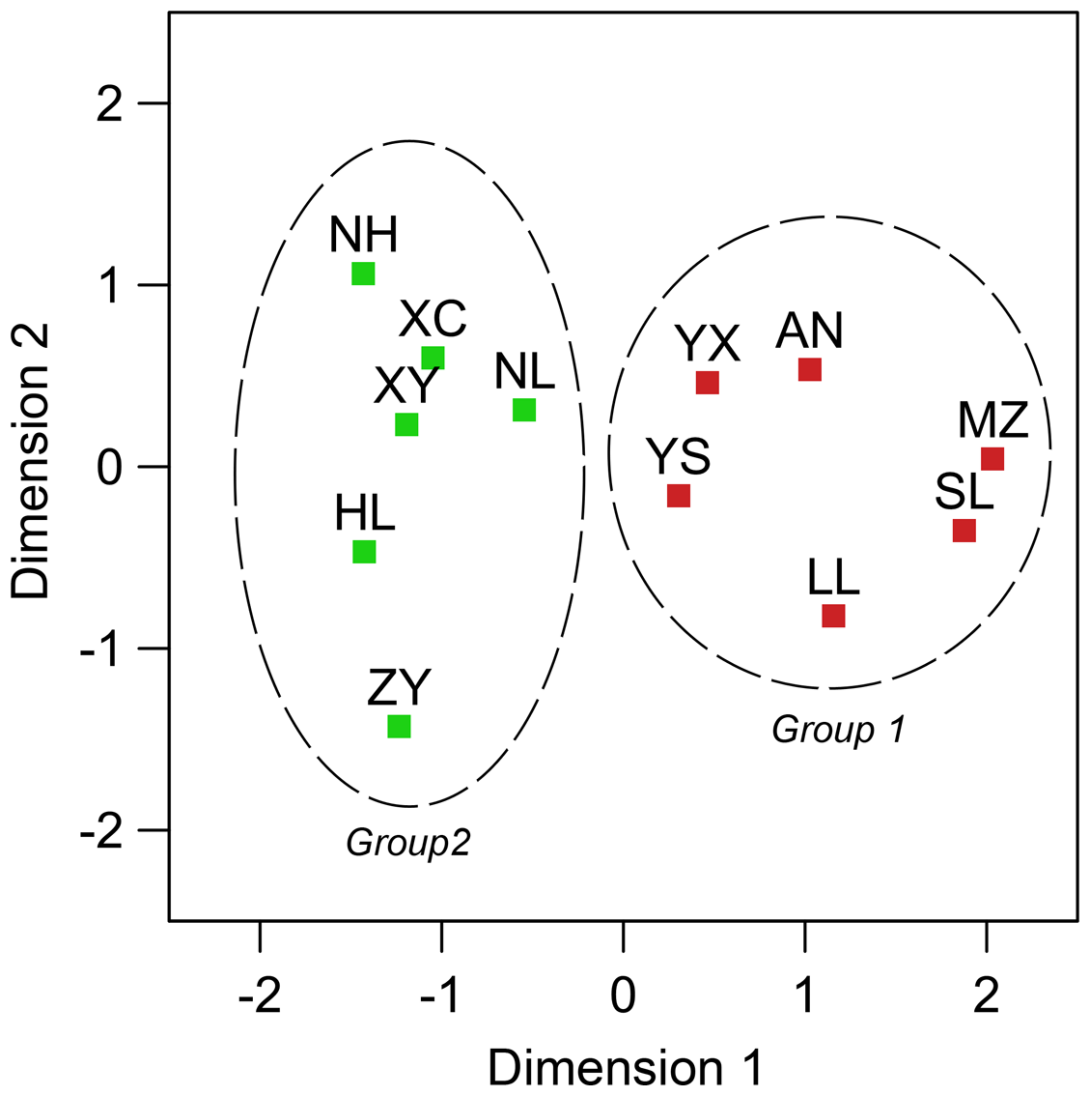
The multidimensional scaling (MDS) plot based on K2P distances of the 12 populations of *T. yunnanensis*, with tentative boundary (dash line) between two population groups.

The SAMOVA analysis determined two population groups, with Anning, Luliang, Mengzi, Shilin, Yuxi, and Yanshan comprising one group (Group 1) and Huili, Nanhua, Ninglang, Xichang, Xiangyun, and Zhanyi forming the other (Group 2) (Fig. 1B). The AMOVA verification detected 32.16% of the total variation among the two population groups (*F*_CT_  = 0.322, *P*<0.01), 13.35% among populations within groups (*F*_SC_  = 0.197, *P*<0.01), and 54.49% within populations (*F*_ST_  = 0.455, *P*<0.01). For the two groups, genetic variation among populations within Group 2 (24.81%) was greater than that within Group 1 (14.34%) (Table 4). Between the two population groups designated by SAMOVA analysis, the K2P distance was 0.011, pairwise *F*st value was 0.344 (*P*<0.001), Nei's average number of differences was 7.718 (*P*<0.001), and the *N*_m_ value was 0.955. The multidimensional scaling (MDS) plot also divided the 12 populations of *T. yunnanensis* into two groups, with populations from Anning, Luliang, Mengzi, Shilin, Yanshan, and Yuxi forming one group (Group 1) and that from Huili, Nanhua, Ninglang, Xichang, Xiangyun, and Zhanyi comprising the other (Group 2) (Fig. 2).

**Table 4:** Results of the AMOVA analysis.

Data division	Source of variation	*d.f.*	Variance component	% of variation	*F*	*P*
Global	Among groups	1	1.241 Va	32.16	*F*_CT_ = 0.322	0.001
	Among populations within groups	10	0.515 Vb	13.35	*F*_SC_ = 0.197	0.000
	Within populations	219	2.103 Vc	54.49	*F*_ST_ = 0.455	0.000
Group 1	Among populations within groups	5	0.374 Va	14.34	*F*_ST_ = 0.143	0.000
	Within populations	108	2.233 Vb	85.66	—	—
Group 2	Among populations within groups	5	0.653 Va	24.81	*F*_ST_ = 0.248	0.000
	Within populations	111	1.978 Vb	75.19	—	—

**Figure 3:**
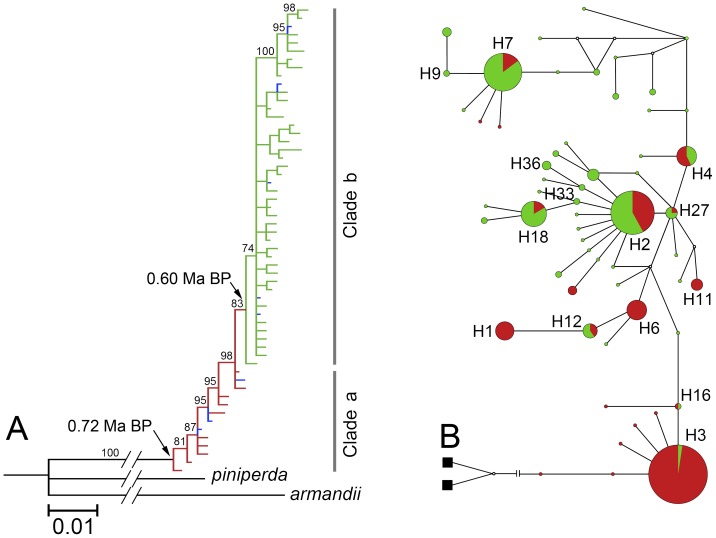
Population genetic structures of *T. yunnanensis*: (A) the Bayesian Inference (BI) tree of 57 haplotypes and outgroups (black terminal) with bootstrap values over 50 (see Fig. S2 for complete haplotype numbers and interior branch bootstrap values) and (B) the median-joining haplotype network denoted with shared haplotypes, the coloration of all three charts were in accordance with the group assignment of both of the SAMOVA analyses, red: Group 1, green: Group 2, blue: admixed.

The BI phylogenetic tree of the 57 haplotypes and two outgroups showed two somewhat clear clades. Clade a directly connected to the outgroups, was predominately occupied by haplotypes found in populations in Group 1 (marked in red), and clade b mostly represents haplotypes derived from populations in Group 2 (marked in green). There were seven haplotypes in the BI tree contained individuals from both of the two population groups (marked in blue) (Fig. 3A; Fig. S2). In general, the BI tree showed a trend that the derivation of haplotypes was from populations in Group 1 to those in Group 2. The *t*_MRCA_ inferred the divergence time between outgroups and clade a as approximately 0.72±0.11 million years before present (Ma BP), and that between clade a and clade b as approximately 0.60±0.10 Ma BP.

The median-joining haplotype network was clearly structured, even with three loops and five median vectors (Fig. 3B). The network showed a clear tendency of evolutionary process from Group 1 to Group 2, and it was noticeable that the middle portion of the network showed a highly admixed pattern of populations from the two population groups and intensively connected private haplotypes, while both ends of this network contained mostly the populations from either group (Fig. 3B).

### Demographic History and IBD

Tajima's *D* values showed positive values in four populations from Anning, Huili, Luliang, and Yuxi, and negative values in the remaining eight populations as well as the global dataset and the populations in both groups. However, among all the negative values, only that of population from Yanshan was flagged a 0.05-level significance (*D* = 1.74, *P*<0.05) (Table 5).

**Table 5:** Statistics of neutrality test (Tajima's *D* and Fu's *F_s_*) and mismatch distribution (SSD and raggedness index), with *P* values in parentheses.

Population	Tajima's *D*	Fu's *F_s_*	SSD	Raggedness Index
Anning	1.78 (0.969)	2.17 (0.835)	0.048 (0.121)	0.138 (0.069)
Huili	2.10 (0.990)	2.09 (0.868)	0.083 (0.094)	0.166 (0.144)
Luliang	0.51 (0.715)	−0.65 (0.387)	0.018 (0.426)	0.037 (0.487)
Mengzi	−0.71 (0.276)	1.39 (0.797)	0.347 (0.000)	0.254 (0.975)
Nanhua	−0.03 (0.529)	−2.36 (0.101)	0.036 (0.112)	0.116 (0.064)
Ninglang	−0.56 (0.313)	−7.08 (0.001)	0.001 (0.906)	0.028 (0.762)
Shilin	−0.03 (0.517)	0.60 (0.650)	0.070 (0.327)	0.132 (0.370)
Xichang	−0.71 (0.273)	−1.54 (0.217)	0.051 (0.330)	0.131 (0.275)
Xiangyun	−0.21 (0.467)	−6.00 (0.004)	0.003 (0.727)	0.018 (0.863)
Yanshan	−1.74 (0.019)	0.48 (0.559)	0.039 (0.276)	0.285 (0.570)
Yuxi	0.11 (0.596)	0.31 (0.574)	0.046 (0.133)	0.094 (0.130)
Zhanyi	−0.08 (0.524)	1.61 (0.803)	0.140 (0.081)	0.322 (0.209)
Group 1	−0.41 (0.404)	−4.02 (0.113)	0.020 (0.225)	0.051 (0.127)
Group 2	−0.93 (0.184)	−25.70 (0.000)	0.005 (0.570)	0.016 (0.634)
Global	−0.68 (0.259)	−25.05 (0.000)	0.004 (0.536)	0.015 (0.438)

Fu's *F_s_* values showed positive values in seven populations of *T. yunnanensis*, from Anning, Huili, Mengzi, Shilin, Yanshan, Yuxi, and Zhanyi. The values showed negative deviation in populations from Luliang, Nanhua, Ninglang, Xichang, and Xiangyun, and significant negative values were detected in populations from Ninglang (*F_s_* = −7.08, *P*<0.001) and Xiangyun (*F_s_* = −6.00, *P*<0.01). Neutrality tests showed significantly large negative values in the global dataset of the 12 populations (*F_s_* = −25.05, *P*<0.001) and that of populations in Groups 2 (*F_s_* = −25.70, *P*<0.001), while the test did not produce significant value of populations in Group 1 (*F_s_* = −4.02, *P*>0.1) (Table 5).

Most of the sum squared deviation (SSD) were not significant except for that of the population from Mengzi (SSD  = 0.35, *P*<0.001), and all raggedness indices were not significant in our analysis (Table 5).

**Figure 4:**
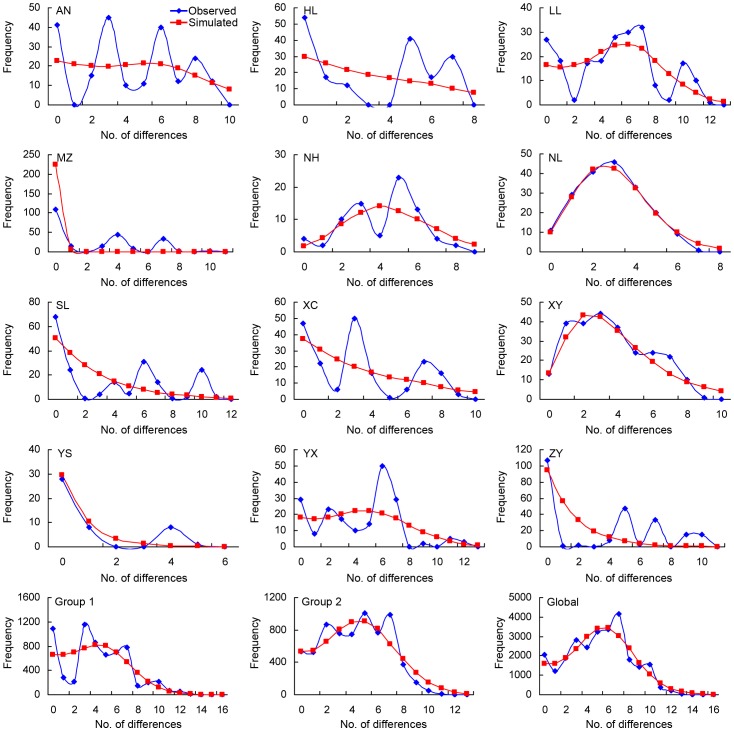
The mismatch distribution of the 12 populations of *T. yunnanensis*, the populations in Group 1, the populations in Group 2, and the global dataset. The blue lines indicated observed mismatch and the red lines indicated the simulation.

The mismatch distribution analysis of the 12 populations of *T. yunnanensis* indicated multimodal pattern in the curves, but the results of the populations from Ninglang and Xiangyun showed a unimodal pattern (Fig. 4). The mismatch distribution curves of the global dataset of the 12 populations and that of the populations in Group 2 showed multimodal pattern in the observed mismatch distribution, but produced a unimodal pattern in the simulated mismatch distribution (Fig. 4A, C). However, the distribution curve of the populations in Group 1 did not show such a pattern (Fig. 4B).

Migration analysis showed that, with both the Bayesian inference and maximum likelihood methods, the effective population size (*θ*) of Group 2 was greater than that of Group 1 at all confident intervals (CI), and the migration rate (*M*) from Group 1 to Group 2 was all greater than that from Group 2 to Group 1 at all CI (Table 6). Large migration rates (>10,000) were detected between populations from Anning to Yuxi (168,291.0) and Zhanyi (10,976.6), Huili to Zhanyi (87,812.7), Luliang to Zhanyi (35,125.1), Mengzi to Shilin (10,384.4), Xichang to Yuxi (67,316.6), and Zhanyi to Yuxi (44,877.7) (Table S3).

**Table 6:** Effective population size (*θ*) and migration rate (*M*) between the two population groups of *T. yunnanensis* based on Bayesian inference and maximum likelihood methods.

Parameter	Bayesian Inference				Maximum Likelihood		
	2.5%	Mode	97.5%	Mean	2.5%	MLE	97.5%
*θ*_Group1_	0.003	0.006	0.011	0.007	0.004	0.006	0.009
*θ*_Group2_	0.014	0.023	0.043	0.027	0.017	0.026	0.034
*M*_Group1→2_	241.3	644.3	990.0	555.6	269.5	814.4	1101.8
*M*_Group2→1_	0.0	279.0	841.3	415.8	19.0	316.3	706.4

**Figure 5:**
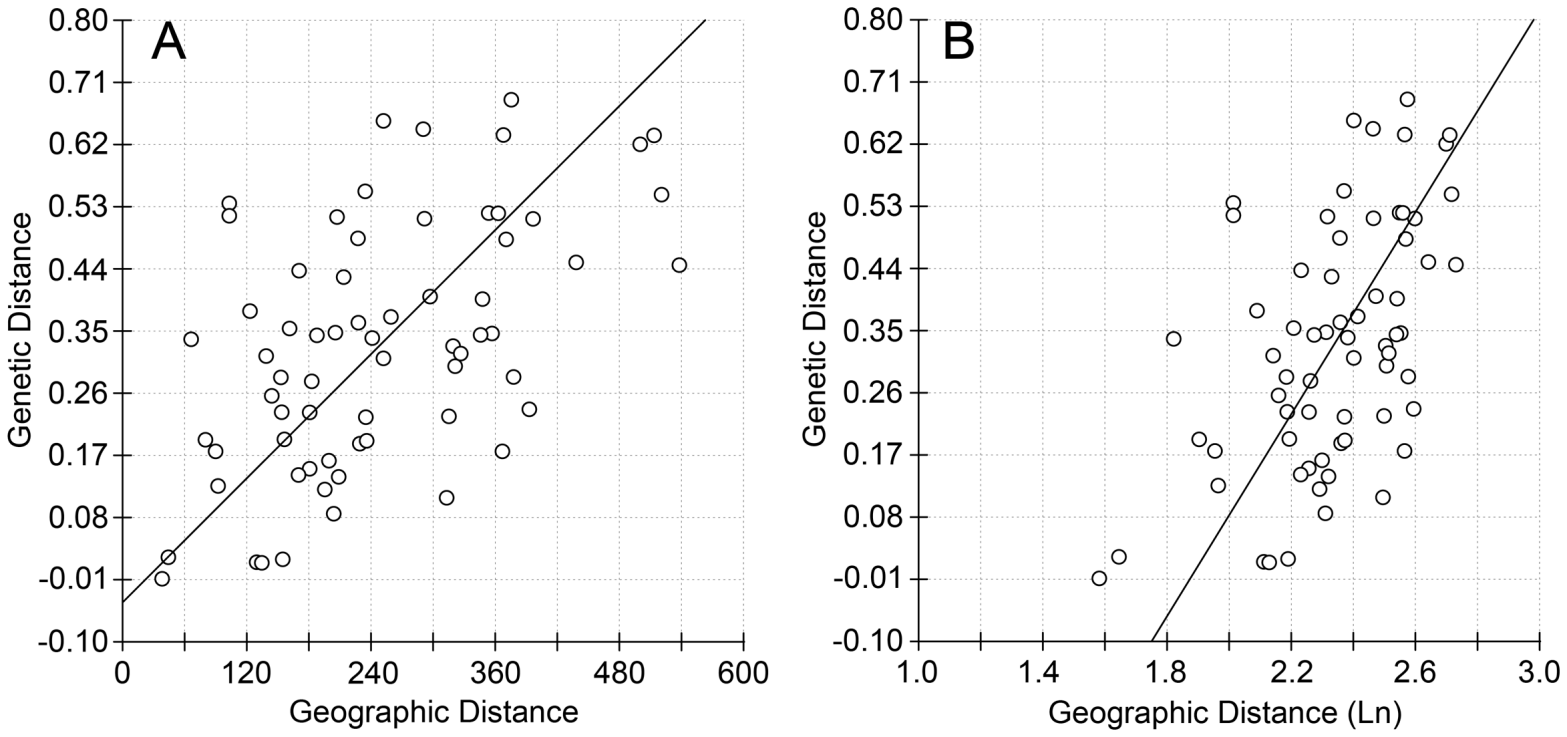
Plots of the isolation-by-distance (IBD) analysis of the 12 population of *T. yunnanensis*, (A) regression between *F*st and geographic distance (*r* = 0.5403, *P*<0.001, with 10,000 iterations), and (B) regression between *F*st and logarithm transformed geographic distance (*r* = 0.5283, *P*<0.001, with 10,000 iterations).

Isolation-by-distance (IBD) was observed for the 12 populations of *T. yunnanensis* (Fig. 5). The correlation between genetic differentiation (*F*st) and the geographic distances was significantly positive (Mantel *r* = 0.540, *P* = 0.001, with 10,000 iterations), and the correlation between *F*st and the logarithm transformed geographic distances was also significantly positive (Mantel *r* = 0.528, *P* = 0.000, with 10,000 iterations). However, no significant positive correlation was found when testing IBD within each of the two population groups using either the geographic distance (for Group 1, Mantel *r* = 0.227, *P* = 0.220; for Group 2, Mantel *r* = 0.405, *P* = 0.094) or the logarithm transformed geographic distance (for Group 1, Mantel *r* = 0.301, *P* = 0.146; for Group 2, Mantel *r* = 0.346, *P* = 0.140).

## Discussion

### Origin of Tomicus yunnanensis

The distribution area of *T. yunnanensis* is currently confined in the Central-Yunnan Altiplano and the adjacent areas of western Yunnan, southwestern Sichuan, and western Guizhou [3]. This particular low-latitudinal area and the longitudinal range-gorge region in its west portion had become a hot spot of species' divergence and origination as a result of the impaction of the Indian plate and the rising of the Himalayas and the Tibetan Plateau [44], [45]. Phylogeny of pine trees inferred that the Yunnan pine, *Pinus yunnanensis*, diverged in 4–3 Ma BP [46], while our analysis suggested that *T. yunnanensis* diverged from its closest ancestor, *T. piniperda*, in 0.72±0.11 Ma BP (Fig. 3A). Moreover, the widespread Eurasian *T. piniperda* feeds on many taxa of Pinaceae [47], [48], but *T. yunnanensis* only feeds only on *P. yunnanensis* [3], [5]. It is interesting to note that another newly described shoot borer, *T. armandii*, which feeds only on the Armand pine, *P. armandii* Franchet, was also found in this region [13]. Both cases implied that host separation could be the driving force of the origin of these two *Tomicus* species.

The sampling range of the present study covered most of the distribution range of *T. yunnanensis* [3], therefore, the results can be used to infer the divergence time and origin area of this beetle. Our phylogenetic reconstruction showed that a haplotype from Mengzi (H19) directly connected to *T. piniperda* of the outgroups and then produced the shared H3 and a few related private haplotypes which are all confined in Central and South Yunnan (Fig. 3A; Fig. S2), indicating the close relationship between these populations and the ancestor. Both the haplotype diversity (*H*) and nucleotide diversity (*π*) of these populations were greater; also Fu's *F_s_* values and the mismatch distribution identified a constant growth pattern over time for these populations (Table 3; Table 5; Fig. 4) [39], [41]. Hence, the authors of the present study tend to believe that Central-Yunnan was the likely area of origin for *T. yunnanensis*.

### Population Divergence

Providing that there was no *Numts* in the dataset and our *Wolbachia* search did not show correlation between the infection rates and the molecular diversity indices of the 12 populations. The authors of the present research tend to attribute the following discussions to phylogenetic and geographic aspects.

*Tomicus yunnanensis* has expanded to different localities [3]. During the course of population expansion, genetic divergence occurred between populations due to isolation caused by geographical barriers and fragmented habitats. Our analyses divided the 12 populations into two groups with 32.16% of the total variance in between (*F*_CT_ = 0.322, *P*<0.01) (Table 4; Fig. 1B), indicating obvious genetic differentiation. Haplotype distribution showed that the shared haplotypes in Group 2 mostly came from part of shared haplotypes in Group 1, and the ratios of private haplotypes (*R*_prv_) of populations in Group 2 were evidently greater than that of Group 1 while the nucleotide diversity (*π*) were much poorer (Fig. 1; Table 3). Therefore, it can be inferred that populations of Group 2 were descendents of some individuals from populations of Group 1.

Haplotypes in Group 1 were directly connected to *T. piniperda* and distributed near the root of the phylogenetic tree and the haplotype network (Fig. 3A, B), indicating the ancestral position of these populations. In comparison, haplotypes in Group 2 were situated in a more evolved position near the end of the tree and the network (Fig. 3A, B). The topology of phylogenetic tree and haplotype network also indicated that the six populations from western Yunnan, southwestern Sichuan, and Zhanyi came from populations from central Yunnan. The most likely process was that some individuals from central Yunnan populations ‘moved’ to novel localities and established their descendent populations there (see below).

The limited K2P distances and *N*_m_ values (Table S1) coupled with significant pairwise *F*st values (Table S2) and significant Mantel test (Fig. 5) indicated obvious genetic differentiation and limited genetic exchange between the two groups. Adult *T. yunnanensis* live in the truck phloem and the shoots of the host, and the limited flying ability only allows them to range from tree to tree for food and mate rather than long distance migration [49]. In Yunnan, the habitats of *P. yunnanensis* between 1,500–2,800 m are fragmented and isolated due to mountainous terrain [6], and the fragmented patches of the pine trees added further barriers to the genetic exchange of the beetle. This is the biological and ecological contribution to obvious genetic differentiation between populations of *T. yunnanensis*, especially between populations from the two groups.

### Demographic History

Our phylogenetic analysis divided the haplotypes into two clades, the BI tree showed that most haplotypes of populations in Group 2 fell into clade b, which was evidently the descendant of clade a containing most haplotypes of populations in Group 1, and the haplotype divergence within populations from clade b was very limited (Fig. 3A). Meanwhile, the migration rate from Group 1 to Group 2 was obviously greater than the other way around (Table 6), indicating a history of population expansion of *T. yunnanensis* after its origination in Central Yunnan. Spatial distribution of haplotypes demonstrated that five shared haplotypes (H2, H4, H7, H18, and H27) in the populations from Huili, Nanhua, Ninglang, Xichang, and Xiangyun all came from the populations in Group 1, while four haplotypes (H2, H7, H18, and H27) were completely shared by the five above-mentioned populations (Fig. 1; Table 2). The reduction trend of haplotypes from the ancestral populations to the descendant populations implied the trace of north- and westward expansion of *T. yunnanensis* from Central Yunnan in the past.

It must be noted that the five above-mentioned populations in Group 2 contained a relatively higher ratio of private haplotypes compared to most populations in Group 1, while haplotypes such as H1, H3, and H6 which are extensively distributed in ancestral populations in Group 1 were completely absent (Fig. 1; Table 2). The median-joining network showed a missing haplotype (median vector) between the ancestral and descendant haplotypes (Fig. 3B), indicating that *T. yunnanensis* underwent a certain kind of selection during its course of expansion, which likely resulted in the loss of haplotypes. It is generally accepted that the founder's effect, release of selective stress, and recent expansion are three major factors which cause negative deviation of evolutionary neutrality and produce a great number of private haplotypes with a star-like pattern in the network [50], [51]. Our neutrality test and mismatch distribution showed that populations like Ninglang and Xiangyun exhibited traces of recent expansion (significant Fu's *F_s_* values, small and insignificant SSD values, and typical unimodal curves of mismatch distribution) (Table 5; Fig. 4). The analyses further implied that recent expansion was not the only explanation of the higher ratio of private haplotypes in the five populations of Group 2.

The five populations are all located in the northern portion of the longitudinal range-gorge region (LRGR) of West Yunnan. Compared to the area of Central and East Yunnan, that particular area has experienced cycles of glacial and interglacial changes in approximately 0.70–0.13 Ma BP [52]–[56]. The cold climate of glacial periods could favour selections and bottlenecks to *T. yunnanensis*, which would likely reduce the genetic diversity, while the warmer climate of interglacial periods could release the stress and allow *T. yunnanensis* to expand quickly, which would likely produce a great number of private haplotypes due to the founder's effect. Our *t*_MRCA_ analysis inferred the divergence of Group 1 and Group 2 in approximately 0.60±0.10 Ma BP, when the northern portion of LRGR was experiencing an interglacial period and the climate was warmer, which allowed *T. yunnanensis* to expand into the area, however, *T. yunnanensis* underwent multiple cold and warm climate oscillations due to glacial and interglacial cycles in that area after the entry. Hence, the authors speculated that the five populations of Group 2 have experienced multiple selective processes as a result of regional climate oscillation, during which the loss of ancestral haplotypes and the generation of private haplotypes occurred.

The population from Zhanyi in Group 2 was different from the other five populations; despite its greater ratio of Group 2 component of the shared haplotypes, the ratio of private haplotypes was considerably lower (only one) (Fig. 1; Table 2). Moreover, Zhanyi is located in East Yunnan, where no obvious climate oscillation occurred in the Pleistocene. Hence, the authors speculated that this particular population should have experienced a different demographic history compared to the other five populations in Group 2. Migration rate analysis showed a large value from Huili to Zhanyi (Table S3), which indicated possible introduction of non-local individuals from Huili to Zhanyi. However, Huili is located 200 km away from Zhanyi with the Wumeng Mountains and the Anning River in between, which constitute geographical barriers. Therefore it is logical to assume that the introduction of *T. yunnanensis* from Huili to Zhanyi should be facilitated by other factors, instead of natural dispersal via the flight of the beetles. Like other borers, *T. yunnanensis* lives in the trunk of its host, which could be easily relocated with untreated wood material [3]. The wood material from the Yunnan pine is commonly used in construction sites, and the transportation of such wood material across the research range could be regarded as a key factor in the relocation of *T. yunnanensis*.

### Conclusion and Implications for Pest Management

The present research suggested that *T. yunnanensis* was originated in central Yunnan in more than 0.7 Ma BP, and expanded into northern, western Yunnan and Sichuan in approximately 0.6 Ma BP. Our analysis implied that regional climate oscillation in the Pleistocene might be responsible for the natural expansion of this beetle, it also the main driving force for shaping the different genetic profiles of these populations. However, the divergence time calculation was solely based on estimated molecular clock, thus the results should be applied with discretion. Moreover, our analysis also identified possible contemporary human-mediated relocation of the beetle from Sichuan to Yunnan.

Like many other pine borers, *T. yunnanensis* could be relocated to novel habitats and go through subsequent establishment. The present research inferred that the expansion of *T. yunnanensis* from central Yunnan to other localities in Yunnan and Sichuan occurred during an interglacial period took place and the regional climate was evidently warmer. Providing its capability of enduring and adapting to climate oscillation, *T. yunnanensis* may expand its range further driven by the warming of global climate change.

Fortunately, the occurrence of *T. yunnanensis* is currently confined to the distribution range of the Yunnan pine due to the restriction of host range [3]. Nevertheless, the Yunnan pine is also found in southern Tibet, western Sichuan, and western Guangxi [6], where *T. yunnanensis* has not been reported to date. Hence, quarantine precautions should be established in advance just to prevent the beetle from spreading into these areas.

## Supporting Information

Figure S1 **Gel images of** ***Wolbachia*** **search for 12 populations of** ***T. yunnanensis*** **.** M: the TaKaRa DL2000 DNA marker, NC: negative control, PC: positive control.
(TIF)

Figure S2 **BI phylogenetic tree of 57 haplotypes of** ***T. yunnanensis*** **and two outgroups.**
(TIF)

Table S1 **The Kimura two-parameter (K2P) distances (below diagonal) and the** ***N*** **_m_ values (above diagonal) between the 12 populations of** ***T. yunnanensis*** **.**
(DOC)

Table S2 **The pairwise** ***F*** **st between populations (below diagonal), Nei's average number of differences within population (diagonal elements), and Nei's average number of differences between populations (above diagonal).**
(DOC)

Table S3 **Migration rates (migrate from row to column) between the 12 populations of** ***T. yunnanensis*** **via maximum likelihood method with 10 short chains consisted of 1,000,000 replications and three long chains consisted of 100,000,000 replications.**
(DOC)
