# Supplementary figures and images for: Origin and Expansion of the Yunnan Shoot Borer, Tomicus yunnanensis (Coleoptera: Scolytinae): A Mixture of Historical Natural Expansion and Contemporary Human-Mediated Relocation
Source: PLoS One. 2014 Nov 5;9(11):e111940. doi: 10.1371/journal.pone.0111940 (PMC4221261; doi:10.1371/journal.pone.0111940)

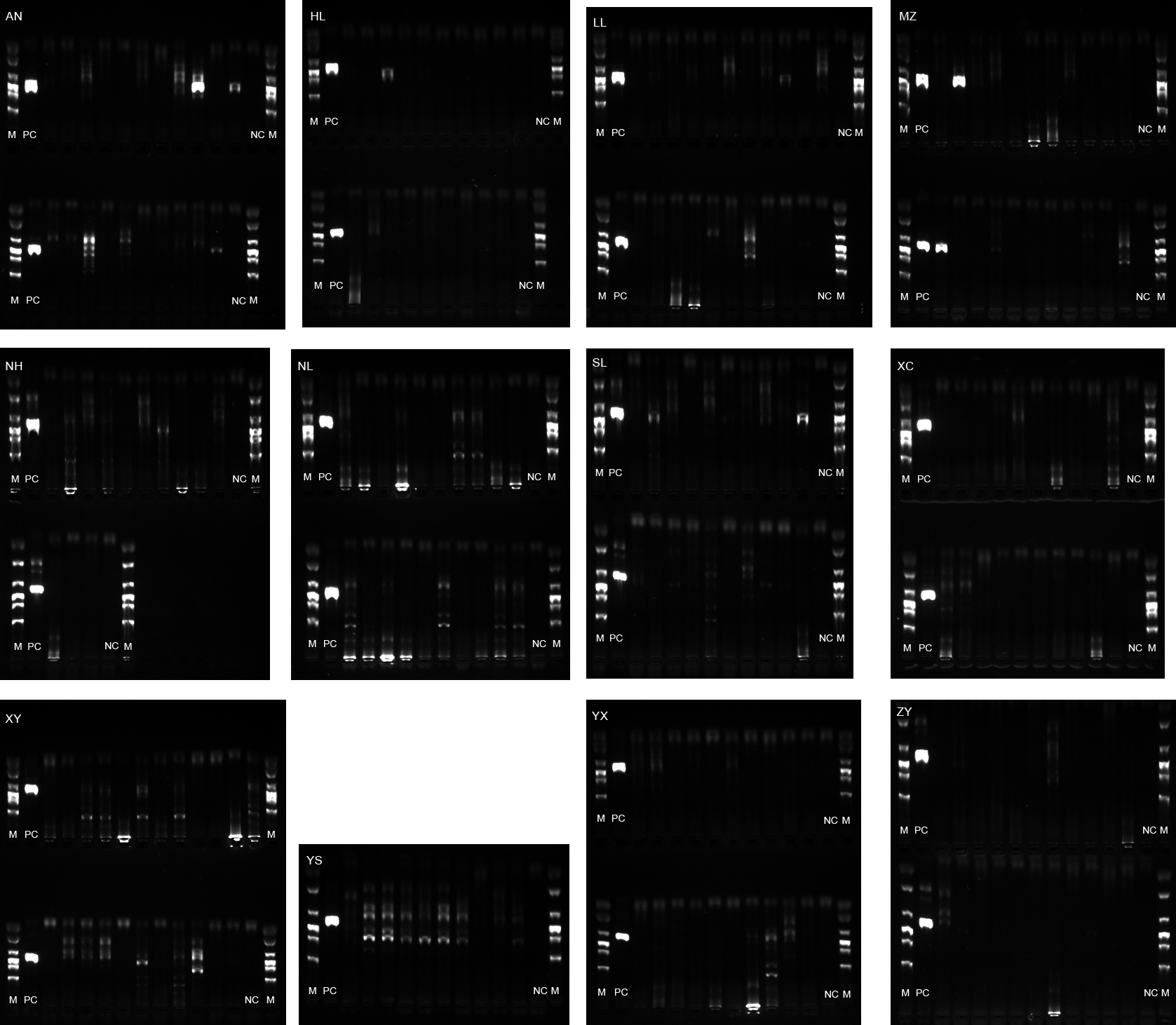

Supplement: Figure S1 — Gel images of Wolbachia search for 12 populations of T. yunnanensis . M: the TaKaRa DL2000 DNA marker, NC: negative control, PC: positive control. (TIF) [file pone.0111940.s001.tif]

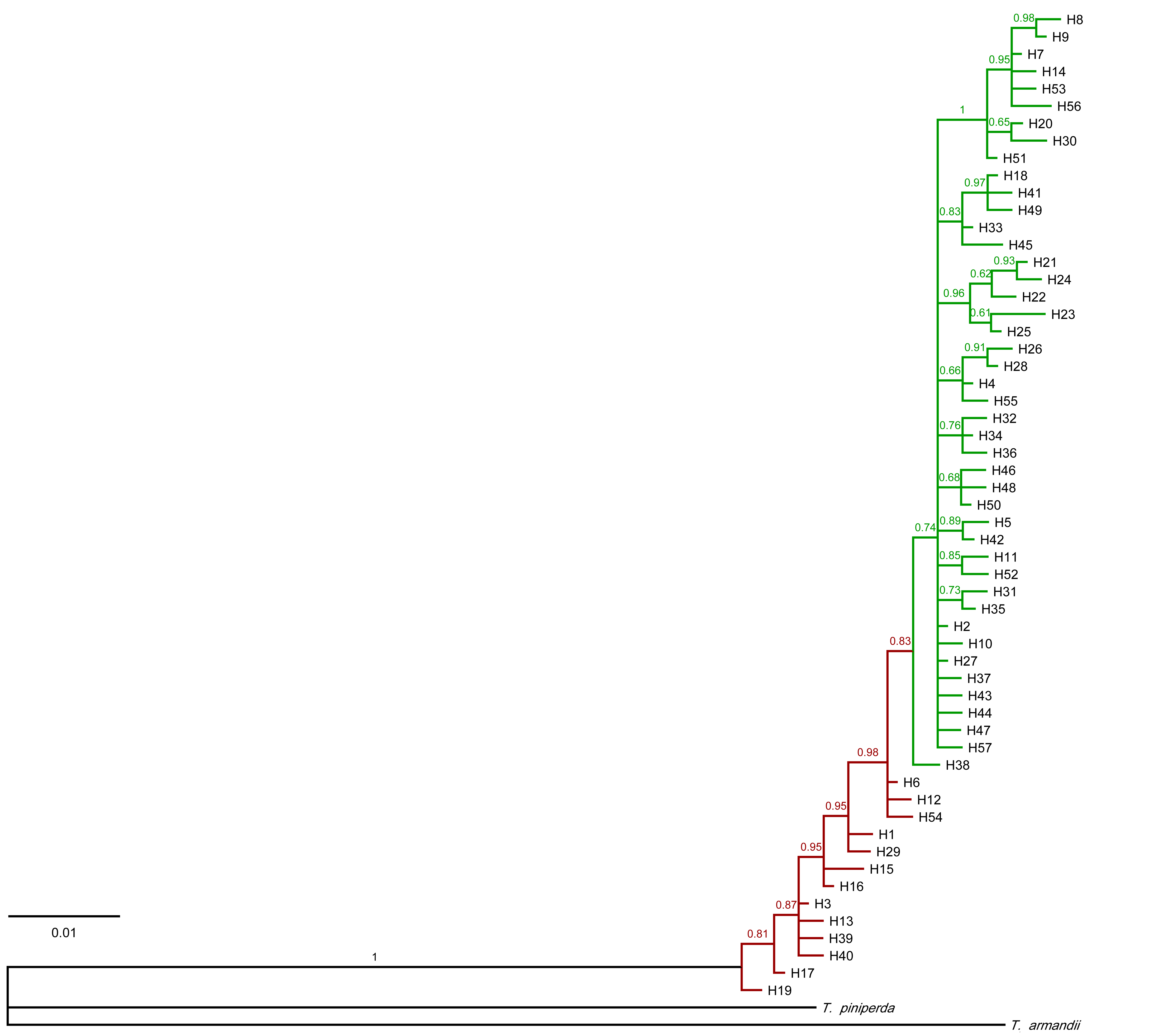

Supplement: Figure S2 — BI phylogenetic tree of 57 haplotypes of T. yunnanensis and two outgroups. (TIF) [file pone.0111940.s002.tif]
