# Supplementary material for: Origin and Expansion of the Yunnan Shoot Borer, Tomicus yunnanensis (Coleoptera: Scolytinae): A Mixture of Historical Natural Expansion and Contemporary Human-Mediated Relocation
Source: PLoS One. 2014 Nov 5;9(11):e111940. doi: 10.1371/journal.pone.0111940 (PMC4221261; doi:10.1371/journal.pone.0111940)
Supplement: Table S1 — The Kimura two-parameter (K2P) distances (below diagonal) and the N m values (above diagonal) between the 12 populations of T. yunnanensis . (DOC) [file pone.0111940.s003.doc]

Table S1: The Kimura two-parameter (K2P) distances (below diagonal) and the *N*_m_ values (above diagonal) between the 12 populations of *T. yunnanensis*.

| **Population** | **AN** | **HL** | **LL** | **MZ** | **NH** | **NL** | **SL** | **XC** | **XY** | **YS** | **YX** | **ZY** |
| --- | --- | --- | --- | --- | --- | --- | --- | --- | --- | --- | --- | --- |
| Anning |  | 0.6683 | 32.7906 | 1.6546 | 0.8204 | 1.1732 | 3.4861 | 0.9435 | 0.9380 | 2.1778 | ∞ | 0.6437 |
| Huili | 0.0109 |  | 0.8818 | 0.2303 | 1.3052 | 1.6536 | 0.4054 | 1.4535 | 3.1057 | 0.4762 | 1.1101 | 3.6537 |
| Luliang | 0.0080 | 0.0111 |  | 2.8251 | 0.9725 | 1.2658 | 21.6901 | 0.9527 | 1.0243 | 3.0355 | 34.5327 | 0.9788 |
| Mengzi | 0.0071 | 0.0146 | 0.0074 |  | 0.2788 | 0.3061 | 25.1035 | 0.2897 | 0.2891 | 0.4352 | 1.0946 | 0.2643 |
| Nanhua | 0.0110 | 0.0082 | 0.0117 | 0.0139 |  | 2.1213 | 0.4713 | 1.7083 | 2.0989 | 0.4603 | 1.2691 | 0.8500 |
| Ninglang | 0.0082 | 0.0064 | 0.0092 | 0.0109 | 0.0067 |  | 0.5361 | 2.0895 | 5.3567 | 0.6223 | 2.3475 | 0.7616 |
| Shilin | 0.0077 | 0.0131 | 0.0077 | 0.0052 | 0.0132 | 0.0104 |  | 0.4607 | 0.4756 | 0.9145 | 2.3476 | 0.4679 |
| Xichang | 0.0093 | 0.0071 | 0.0106 | 0.0122 | 0.0075 | 0.0059 | 0.0120 |  | 4.1075 | 0.4134 | 1.6128 | 0.7506 |
| Xiangyun | 0.0098 | 0.0065 | 0.0107 | 0.0131 | 0.0075 | 0.0056 | 0.0123 | 0.0061 |  | 0.6130 | 1.7191 | 1.0762 |
| Yanshan | 0.0057 | 0.0079 | 0.0064 | 0.0064 | 0.0089 | 0.0059 | 0.0067 | 0.0082 | 0.0074 |  | 2.5835 | 0.5331 |
| Yuxi | 0.0071 | 0.0092 | 0.0081 | 0.0081 | 0.0097 | 0.0071 | 0.0083 | 0.0081 | 0.0084 | 0.0056 |  | 0.9548 |
| Zhanyi | 0.0116 | 0.0065 | 0.0111 | 0.0143 | 0.0099 | 0.0087 | 0.0128 | 0.0093 | 0.0086 | 0.0083 | 0.0101 |  |
