# Supplementary material for: Origin and Expansion of the Yunnan Shoot Borer, Tomicus yunnanensis (Coleoptera: Scolytinae): A Mixture of Historical Natural Expansion and Contemporary Human-Mediated Relocation
Source: PLoS One. 2014 Nov 5;9(11):e111940. doi: 10.1371/journal.pone.0111940 (PMC4221261; doi:10.1371/journal.pone.0111940)
Supplement: Table S2 — The pairwise F st between populations (below diagonal), Nei's average number of differences within population (diagonal elements), and Nei's average number of differences between populations (above diagonal). (DOC) [file pone.0111940.s004.doc]

Table S2: The pairwise *F*st between populations (below diagonal), Nei’s average number of differences within population (diagonal elements), and Nei’s average number of differences between populations (above diagonal).

| **Population** | **AN** | **HL** | **LL** | **MZ** | **NH** | **NL** | **SL** | **XC** | **XY** | **YS** | **YX** | **ZY** |
| --- | --- | --- | --- | --- | --- | --- | --- | --- | --- | --- | --- | --- |
| Anning | **5.0846** | 7.8617** | 5.7668 | 5.1214** | 7.9145** | 5.9133** | 5.5214* | 6.7277** | 7.0399** | 4.0886 | 5.1142 | 8.3715** |
| Huili | 0.4280** | **3.8791** | 7.9983** | 10.4926** | 5.8959** | 4.5996** | 9.4478** | 5.0891** | 4.6455* | 5.6659** | 6.6201** | 4.6805* |
| Luliang | 0.0150 | 0.3619** | **6.2757** | 5.3284** | 8.4395** | 6.6218** | 5.5490 | 7.6226** | 7.7040** | 4.6019 | 5.8405 | 8.0108** |
| Mengzi | 0.2321** | 0.6847** | 0.1504** | **2.7981** | 9.9858** | 7.8675** | 3.7525 | 8.8102** | 9.4675** | 4.6336** | 5.8181** | 10.3314** |
| Nanhua | 0.3787** | 0.2770** | 0.3396** | 0.6420** | **4.7075** | 4.8181** | 9.5421** | 5.3838** | 5.4333** | 6.3899** | 6.9612** | 7.1561** |
| Ninglang | 0.2988** | 0.2322** | 0.2832** | 0.6203** | 0.1908** | **3.1901** | 7.4988** | 4.2667** | 4.0015* | 4.2293** | 5.1102** | 6.2647** |
| Shilin | 0.1254* | 0.5523** | 0.0223 | 0.0195 | 0.5148** | 0.4826** | **4.5702** | 8.6180** | 8.8927** | 4.8197* | 5.9472* | 9.2174** |
| Xichang | 0.3464** | 0.2560** | 0.3442** | 0.6332** | 0.2264** | 0.1931** | 0.5204** | **3.6955** | 4.3857* | 5.9334** | 5.8500** | 6.7126** |
| Xiangyun | 0.3477** | 0.1387* | 0.3280** | 0.6337** | 0.1924** | 0.0854** | 0.5125** | 0.1085* | **4.1182** | 5.3480** | 6.0215** | 6.1980** |
| Yanshan | 0.1867* | 0.5122** | 0.1414* | 0.5346** | 0.5207** | 0.4455** | 0.3535** | 0.5474** | 0.4493** | **1.1091** | 4.0358 | 5.9863** |
| Yuxi | -0.0090 | 0.3105** | 0.0143 | 0.3136** | 0.2826** | 0.1756** | 0.1756* | 0.2367** | 0.2253** | 0.1622* | **5.2357** | 7.2873** |
| Zhanyi | 0.4372** | 0.1204* | 0.3381** | 0.6542** | 0.3704** | 0.3963** | 0.5166** | 0.3998** | 0.3172** | 0.4840** | 0.3437** | **4.3476** |

* Significant at the 0.05 level.

** Significant at the 0.01 level.
