# Supplementary material for: Origin and Expansion of the Yunnan Shoot Borer, Tomicus yunnanensis (Coleoptera: Scolytinae): A Mixture of Historical Natural Expansion and Contemporary Human-Mediated Relocation
Source: PLoS One. 2014 Nov 5;9(11):e111940. doi: 10.1371/journal.pone.0111940 (PMC4221261; doi:10.1371/journal.pone.0111940)
Supplement: Table S3 — Migration rates (migrate from row to column) between the 12 populations of T. yunnanensis via maximum likelihood method with 10 short chains consisted of 1,000,000 replications and three long chains consisted of 100,000,000 replications. (DOC) [file pone.0111940.s005.doc]

Table S3: Migration rates (migrate from row to column) between the 12 populations of *T. yunnanensis* via maximum likelihood method with 10 short chains consisted of 1,000,000 replications and three long chains consisted of 100,000,000 replications.

| **Population** | **AN** | **HL** | **LL** | **MZ** | **NH** | **NL** | **SL** | **XC** | **XY** | **YS** | **YX** | **ZY** |
| --- | --- | --- | --- | --- | --- | --- | --- | --- | --- | --- | --- | --- |
| Anning |  | 0.0 | 0.0 | 0.0 | 0.0 | 0.0 | 0.0 | 0.0 | 0.0 | 0.0 | 0.0 | 0.0 |
| Huili | 0.0 |  | 0.0 | 0.0 | 0.0 | 0.0 | 0.0 | 0.0 | 0.0 | 0.0 | 0.0 | 6036.8 |
| Luliang | 1072.9 | 0.0 |  | 2048.3 | 0.0 | 0.0 | 1463.1 | 0.0 | 0.0 | 0.0 | 0.0 | 0.0 |
| Mengzi | 0.0 | 0.0 | 0.0 |  | 487.5 | 0.0 | 812.6 | 0.0 | 0.0 | 1950.2 | 0.0 | 0.0 |
| Nanhua | 0.0 | 0.0 | 285.1 | 0.0 |  | 0.0 | 0.0 | 0.0 | 0.0 | 0.0 | 213.8 | 0.0 |
| Ninglang | 545.2 | 204.4 | 204.4 | 0.0 | 0.0 |  | 0.0 | 0.0 | 0.0 | 0.0 | 0.0 | 0.0 |
| Shilin | 0.0 | 0.0 | 1730.7 | 10384.4 | 0.0 | 0.0 |  | 0.0 | 0.0 | 865.4 | 0.0 | 0.0 |
| Xichang | 0.0 | 0.0 | 1092.4 | 0.0 | 0.0 | 0.0 | 0.0 |  | 0.0 | 0.0 | 546.2 | 2184.8 |
| Xiangyun | 0.0 | 0.0 | 0.0 | 0.0 | 0.0 | 633.9 | 0.0 | 1901.7 |  | 0.0 | 0.0 | 0.0 |
| Yanshan | 7161.5 | 0.0 | 0.0 | 0.0 | 0.0 | 0.0 | 0.0 | 0.0 | 0.0 |  | 0.0 | 0.0 |
| Yuxi | 168291.0 | 0.0 | 0.0 | 0.0 | 0.0 | 0.0 | 0.0 | 67316.6 | 0.0 | 0.0 |  | 44877.7 |
| Zhanyi | 10976.6 | 87812.7 | 35125.1 | 0.0 | 0.0 | 0.0 | 0.0 | 0.0 | 0.0 | 0.0 | 0.0 |  |
